# Wide-spread dispersal in a deep-sea brooding polychaete: the role of natural history collections in assessing the distribution in quill worms (Onuphidae, Annelida)

**DOI:** 10.1186/s12983-023-00520-0

**Published:** 2024-01-18

**Authors:** Nataliya Budaeva, Stefanie Agne, Pedro A. Ribeiro, Nicolas Straube, Michaela Preick, Michael Hofreiter

**Affiliations:** 1 Department of Natural History, University Museum of Bergen, University of Bergen, Allégaten 41, 5007 Bergen, Norway; 2 Evolutionary Adaptive Genomics, Department of Mathematics and Natural Sciences, Institute for Biochemistry and Biology, University of Potsdam, Potsdam, Germany; 3 Department of Biological Sciences and Centre for Deep-Sea Research, University of Bergen, Thormøhlens Gate 53B, 5006 Bergen, Norway

**Keywords:** Polychaetes, *Hyalinoecia*, Cosmopolitan distribution, Archival DNA, Target capture

## Abstract

**Background:**

Modern integrative taxonomy-based annelid species descriptions are detailed combining morphological data and, since the last decades, also molecular information. Historic species descriptions are often comparatively brief lacking such detail. Adoptions of species names from western literature in the past led to the assumption of cosmopolitan ranges for many species, which, in many cases, were later found to include cryptic or pseudocryptic lineages with subtle morphological differences. Natural history collections and databases can aid in assessing the geographic ranges of species but depend on correct species identification. Obtaining DNA sequence information from wet-collection museum specimens of marine annelids is often impeded by the use of  formaldehyde and/or long-term storage in ethanol resulting in DNA degradation and cross-linking.

**Results:**

The application of ancient DNA extraction methodology in combination with single-stranded DNA library preparation and target gene capture resulted in successful sequencing of a 110-year-old collection specimen of quill worms. Furthermore, a 40-year-old specimen of quill worms was successfully sequenced using a standard extraction protocol for modern samples, PCR and Sanger sequencing. Our study presents the first molecular analysis of *Hyalinoecia* species including the previously known species *Hyalinoecia robusta*, *H. tubicloa*, *H. artifex*, and *H. longibranchiata,* and a potentially undescribed species from equatorial western Africa morphologically indistinguishable from *H. tubicola*. The study also investigates the distribution of these five *Hyalinoecia* species. Reassessing the distribution of *H. robusta* reveals a geographical range covering both the Atlantic and the Indian Oceans as indicated by molecular data obtained from recent and historical specimens.

**Conclusion:**

Our results represent an example of a very wide geographical distribution of a brooding deep-sea annelid with a complex reproduction strategy and seemingly very limited dispersal capacity of its offspring, and highlights the importance of molecular information from museum specimens for integrative annelid taxonomy and biogeography.

**Supplementary Information:**

The online version contains supplementary material available at 10.1186/s12983-023-00520-0.

## Background

Species with cosmopolitan (i.e., occurring in at least two major ocean basins) geographical ranges were commonly reported for annelids throughout the nineteenth and twentieth centuries. The reasons for this were often limitations of the original, centuries-old descriptions, subsequent global use of species names published in European literature, lack of critical assessment of variation in diagnostic characters and unavailability of molecular data (see review in [1]). Many of these supposedly widely distributed species were subsequently shown to be similar species with minute morphological differences or species complexes comprising a number of genetic lineages with nearly identical morphology [2–5]. A number of invasive annelid species are known to have extremely wide geographical ranges due to human-induced translocations [6–9]. Application of molecular data confirmed a few cases of wide distributions of supposed non-invasive species. Meißner et al. [10] reported *Pholoe longa* (O.F. Müller, 1776) [11], a shallow subtidal scale worm common in the Canadian Arctic with records in both the North Atlantic (off Nova Scotia) and in the North Pacific (off Alaska). Several annelid species associated with chemosynthesis-based ecosystems (vent and seep) were reported across long distances in the Antarctic, the Indian and the Pacific Oceans [12] or across the Atlantic Ocean from pole to pole [13, 14]. Guggolz et al. [15] reported several deep-sea lineages in the spionid genera *Prionospio* and *Aurospio* occurring in both, Atlantic and Pacific Oceans. Recently, three more species of abyssal annelids, one pilargid, *Sigambra magnuncus* Paterson & Glover, 2000 [16], and two goniadids, *Progoniada regularis* Hartman, 1965 [17] and *Bathyglycinde profunda* (Hartman & Fauchald, 1971) [18], were confirmed to have a pan-oceanic distribution based on COI data [19]. In the same study [19], a vast geographical range spanning from the East to the West Pacific was reported for a spionid species *Spiophanes pacificus* Meißner, Schwentner and Fiege, 2023 [19]. Nevertheless, such records of wide geographical ranges supported by molecular data remain rare.

Hutchings and Kupriyanova [1] emphasize the value of databases and museum collections in assessing geographical ranges of marine species in general. Natural history collections house numerous easily accessible specimens with information about their morphology and sampling locality, providing the possibility to further reveal their phylogenetic position and true geographic distribution. However, 100 years of use of formalin in annelid preservation in natural history museum collections created an obstacle in the use of historical materials for molecular analyses. Formalin became popular in medical and histological work at the very end of the nineteenth century and was used as the main preservation fluid in most wet collections of soft-bodied organisms throughout the twentieth century [20] until the inclusion of DNA sequence information in taxonomy by the end of the twentieth century. Formalin-preserved samples pose a challenge for inclusion in molecular studies. Standard DNA extraction protocols often fail, and PCR amplification and sequencing are impeded due to cross-linking forming methylene bridges between proteins, between proteins and nucleic acids and acid-driven hydrolytic fragmentation of nucleic acids caused by unbuffered formalin solutions [21]. Several methods bypassing the effect of formalin on DNA preservation in biological samples were developed in recent years [22, 23]. Following formalin fixation, samples are often preserved in Ethanol, which causes an increased level of DNA fragmentation owing to the solvent's hydrolytic effect [24]. Combining ancient DNA extraction protocols with single-stranded DNA library preparation and target gene capture were shown to be effective approaches for obtaining DNA sequence information of archival collection specimens, including from formalin-fixed wet-collection material [25, 26].

The genus *Hyalinoecia*, the so-called quill worms, comprises approximately 20 valid species [27] and is known from subtidal to lower bathyal depths with a global distribution except for the Arctic Oceans [28]. The worms have a peculiar lifestyle inhabiting robust and transparent tubes that they carry along while crawling on the sea floor in search for food [29, 30]. The tubes are of organic material, secreted by their inhabitants and are composed of a unique substance, onuphic acid [31]. In *Hyalinoecia*, the tubes are cylindrical in cross-section with several valves at both openings, while in its supposed sister genus, *Leptoecia* Chamberlin, 1919 [32], they are mostly flattened and lack internal valves. The tubes of quill worms are unique within annelids. They are reported to have different degrees of curvature and thickness, but these characters are hard to quantify, thus the tube morphology is not commonly used for species discrimination.

Most *Hyalinoecia* species are known from their type localities only and their documented distribution ranges are restricted due to the low number of known records. Others, such as *Hyalinoecia tubicola* (O.F. Müller, 1776) [11], were reported worldwide possibly due to misidentifications or the use of the oldest known species name for worms with very unusual and remarkable tubes.

*Hyalinoecia robusta* Southward, 1977 [33] is a deep-sea species originally described from depths of 1500–2300 m in the Bay of Biscay with an additional record from off La Gomera, Canary Islands at 1000 m [33]. It has been subsequently reported from other East Atlantic sites off Morocco and the Western Sahara at depths of 603–1790 m ([34–36]; off Sierra Leone and Ghana at depths of 1260–2100 m [37]; off Equatorial Guinea and Angola at depths of 260–650 m [38] and very recently close to the type locality at a depth of 1500 m [39]. Moreover, [37] reported 29 specimens from four deep-water stations (1160–2000 m) in Indonesia; however, no detailed morphological description of that material was given. Despite a relatively wide distribution reported in the East Atlantic slope areas, Arias and Paxton [39] suggested that *H. robusta* had a very restricted geographical range in the Northeast Atlantic corroborated by its brooding reproductive strategy assumingly limiting dispersal potential, a suggestion challenging the records in [37]. Therefore, they suggested that *H. robusta* may represent a species complex outside the North-East Atlantic [9, 36].

The University Museum of Bergen (University of Bergen, Norway) holds a collection of marine invertebrates sampled during the RV *Michael Sars* North Atlantic Deep-Sea Expedition, 1910 [40]. The expedition obtained benthic trawl samples at water depths down to 5000 m in the areas of western and southern Europe, north-western Africa, and then crossed the Atlantic Ocean toward Newfoundland and returned to Europe. Numerous *Hyalinoecia* specimens were collected during this expedition in the eastern Atlantic from shelf to continental depths (200–2000 m deep), all identified as *H. tubicola* by James A. Grieg. Notably, the specimens from the deepest stations were significantly larger in size inhabiting very robust and nearly straight tubes. Furthermore, a number of large specimens were collected by the EAF-Nansen programme at the slope depths of Western African coast, off Nigeria in 2005. These specimens were preserved in 96% ethanol but were probably removed from their characteristic tubes on board of the vessel and were not labelled as *Hyalinoecia* in the museum collection until they were identified using DNA barcoding during the “Marine Invertebrates of Western Africa—MIWA” initiative https://miwa.w.uib.no.

In the present study we combine all available records of *Hyalinoecia robusta* with molecular data obtained from recently collected and historical specimens across their geographical and vertical ranges to review the distribution of this species. We also provide an updated species description of *H. robusta* based on examination of the type and non-type material. Furthermore, we present a robust phylogenetic background for delimitation of the three *Hyalinoecia* species inhabiting the North Atlantic Ocean*: Hyalinecia robusta*, *H. tubicola* and *H. artifex* Verrill, 1880 [41].

## Results

### Molecular results

Historical specimen ZMBN 153529: 1,870,201 raw reads were available after test-sequencing, 4,294,323 raw reads were sequenced from the target captured libraries [25]. Overall, 1,111,700 (test-sequencing) and 3,725,957 (target capture) trimmed and filtered reads were used for obtaining consensus sequences. Five of the six targeted marker sequences were successfully reconstructed, i.e., mitochondrial 16S rRNA (16S) and nuclear internal transcribed spacers ITS1, ITS2, 28S rRNA (28S), and 18S rRNA (18S). We were not able to recover sequence information from the COI gene. Filtered reads were used to map against the target genes, which allowed around 45% (ITS1), 75% (ITS2), 92% (16S) and 100% (18S and 28S) completeness of the genes.

**Fig. 1:**
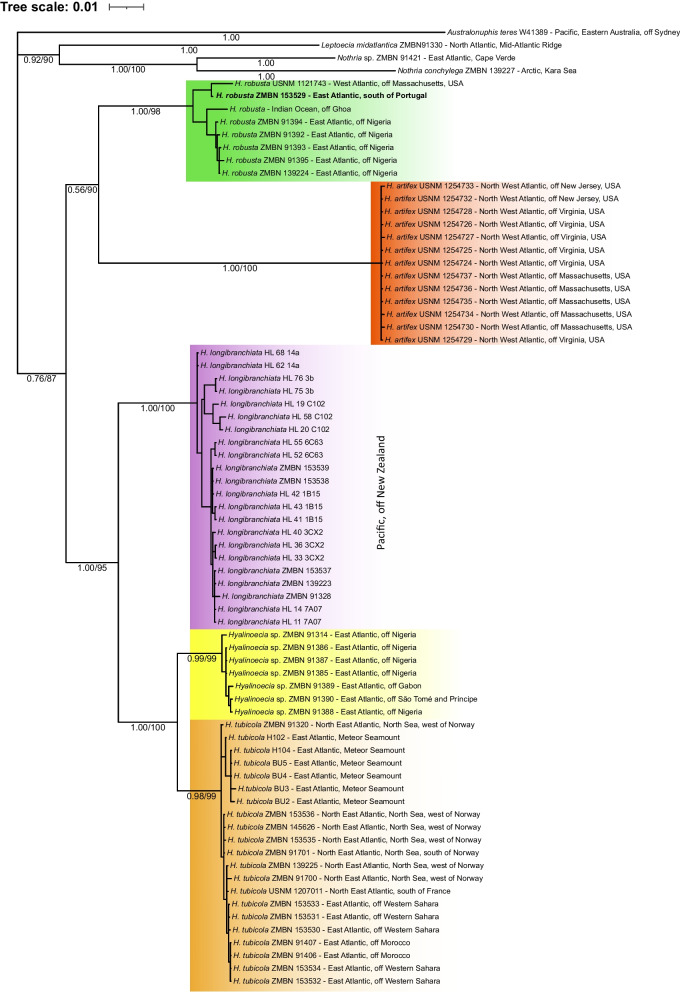
Phylogram summarizing the Bayesian and maximum likelihood analyses of *Hyalinoecia* based on the following genes: 16S, 18S, 28S, COI, ITS1, and ITS2. Only 16S data were available for *H. artifex*. *Australonuphis teres*, *Leptoecia midatlantica*, *Nothria* sp. and *Nothria conchylega* were chosen as outgroups. Numbers on branches indicate posterior probabilities/bootstrap values (%). Historical specimen of *H. robusta* is shown in bold

**Fig. 2:**
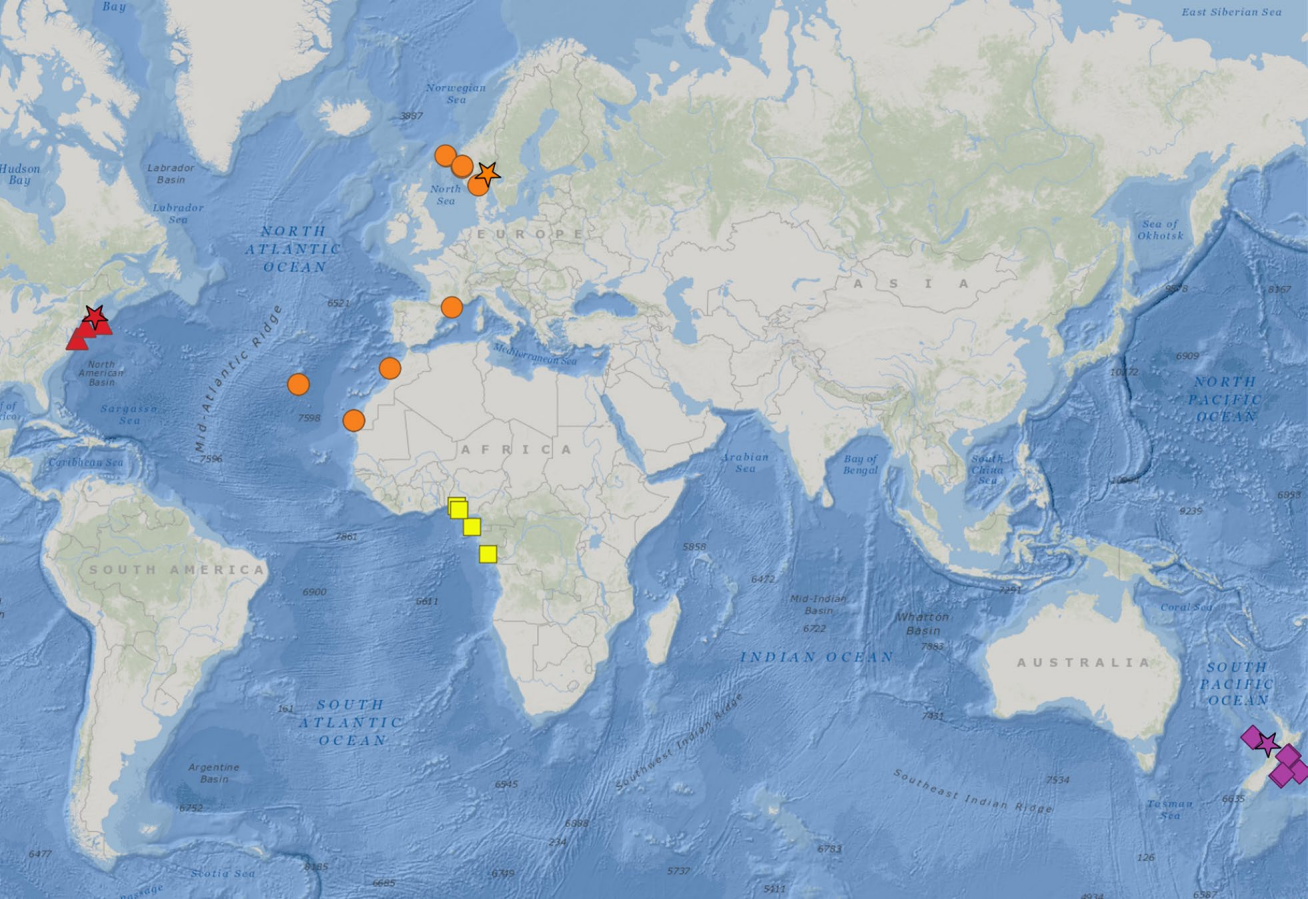
Distribution of *Hyalinoecia* species used in the present study, records are based on vouchered specimens with associated molecular information. Each color represents a species: yellow squares = *Hyalinoecia* sp.; purple rhombuses = *H. longibranchiata*; red triangles = *H. artifex*; orange circles = *H. tubicola*. Stars indicate type localities of respective species. Numbers represent depths. Distribution of *H. robusta* is shown on Fig. 5

The combined dataset had 4926 aligned positions including gaps (658 for COI, 957 for 16S, 1770 for 18S, 518 for ITS1, 362 for ITS2, and 661 for 28S). There was high congruence between the trees obtained with the Bayesian and Maximum Likelihood approaches (Fig. 1). In both analyses the following clades were obtained: *Hyalinoecia* is monophyletic (PP = 0.76, BP = 87). Five highly supported clades (PP = 0.98–1.00, BP = 98–100) corresponding to the four species as well as a single undescribed lineage, *Hyalinoecia* sp., were recovered. *Hyalinoecia tubicola* was reported from shelf depths in the Nordic Seas, the Meteor Seamount and north-western Africa (Morocco and West Sahara). Its sister clade, *Hyalinoecia* sp., morphologically indistinguishable from *H. tubicola*, was reported from the shelf areas of equatorial western Africa (off Nigeria, São Tomé and Príncipe, and Gabon) (Fig. 1). The *Hyalinoecia tubicola* / *Hyalinoecia* sp. complex was sister to *H. longibranchiata* McIntosh, 1885 [42] from the deep Southern Pacific (PP = 1.00, BP = 95). *Hyalinoecia artifex* from the western Atlantic is sister (PP = 0.56, BP = 90) to *H. robusta* which combines specimens collected in several deep-sea localities in the Atlantic and the Indian oceans: off Massachusetts, USA, south of Portugal, off Nigeria and off Goa, India (Figs. 2 and 5).

### Distances

Within species p-distances varied between 0.06 and 2.7% in COI; 0% and 0.9% in 16S; 0% and 0.11% in ITS1; 0% and 0.42% in ITS2; and 0% and 0.1% in 28S. No within-species variation was reported in 18S sequences. In *H. artifex*, the distances were estimated for 16S only due to lack of data for other markers. *Hyalinoecia robusta* showed the highest values of intraspecific sequence divergence in all analyzed markers except ITS1 (Additional file 1).

Between species p-distances were largest in mitochondrial markers: 9.6–18.1% in COI and 6.2–11.0% in 16S, except for the very low p-distance (0.4%) between *H. tubicola* and *Hyalinoecia* sp. in the sequenced fragment of 16S. The p-distances between the species in the nuclear internal transcribed spacers varied between 1.4 and 7.5% in ITS1 and between 2.6 and 6.5% in ITS2. The most conservative markers were the nuclear rRNA with 0.6–2.3% p-distances in 28S and nearly no differences between the 18S sequences (Additional file 1).

### Taxonomic account

Below we provide the redescription of *H. robusta* based on the examined type material and the specimens from the eastern and the western Atlantic.

### *Hyalinoecia robusta* Southward, 1977 (Figs. 3 and 4)

**Fig. 3:**
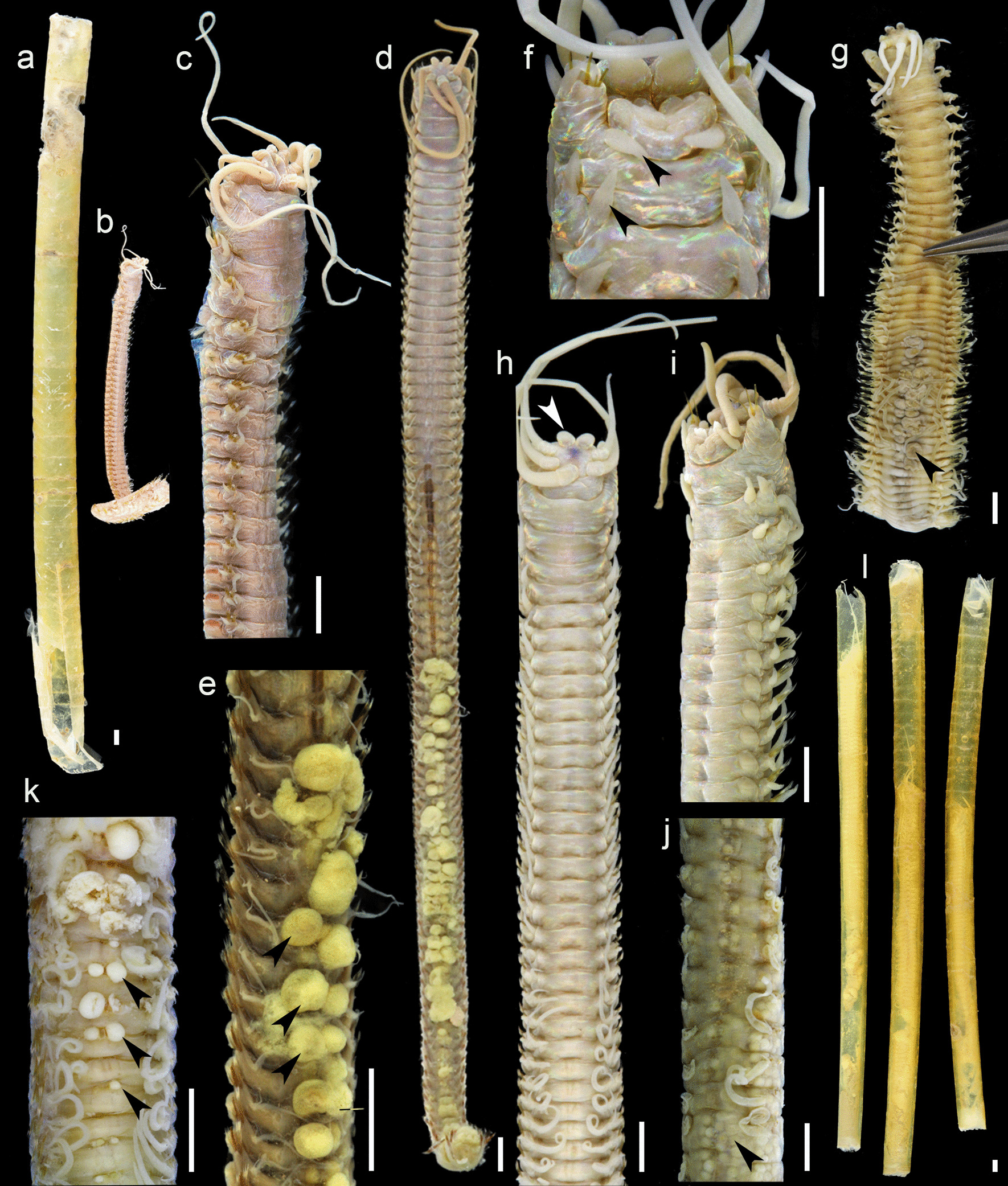
*Hyalinoecia robusta*, **a** tube of the holotype BMNH 1975.194; **b** holotype BMNH 1975.194; **c** the same, enlarged anterior fragment; **d** ZMBN 156645, dorsal view; **e** the same, posterior segments, arrowheads indicate spermaducal papillae; **f** ZMBN 29500, anterior region, ventral view, arrowheads indicate elongated ventral parapodial cirri; **g** ZMBN 156643, anterior fragment, dorsal view, arrowhead indicates spermaducal papillae; **h** ZMBN 153529, anterior region, dorsal view, arrowhead indicates frontal lips; **i** ZMBN 156646, anterior region, latero-ventral view; **j** the same, posterior region, arrowhead indicates oocytes in the body cavity; **k** ZMBN 29500, median region, arrowheads indicate spermaducal papillae; **l** ZMBN 29500, tubes with worms inside. Scale bars—2 mm

**Fig. 4:**
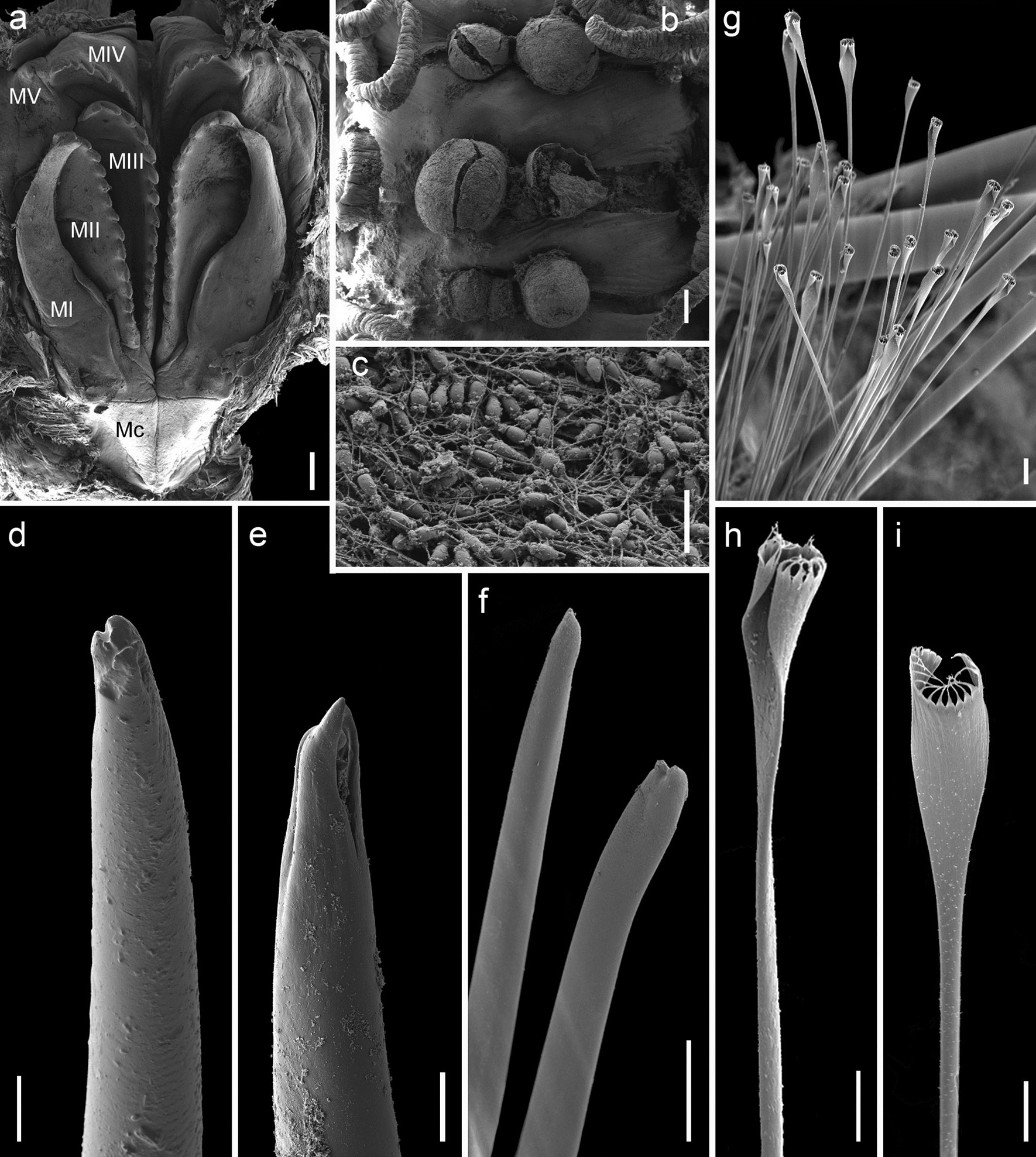
*Hyalinoecia robusta*, **a** ZMBN 29500, maxillary apparatus; **b** ZMBN 29500, dorsal papillae; **c** the same, elongated spermatozoa inside papillae; **d**, **e** ZMBN 29500, simple falcigers from chaetiger 1; **f** ZMBN 91392, intrafascicular bidentate hooks from midbody; **g** the same, pectinate chaetae from midbody; **h**, **i** the same, enlarged pectinate chaetae from midbody. Mc – maxillary carriers; MI–MV – maxillary plates.  Scale bars: **a**, **b**—200 µm; **c**, **h**, **i**—10 µm; **d**, **e**, **g**—20 µm; **f**—100 µm

*Hyalinoecia robusta* Southward, 1977 [33]: 175–180, pl. 1, figs a–j; pl. 2, figs a–b.

*Hyalinoecia robusta* Rozenfeldt 1982 [36]: 47–48; Hartmann-Schröder 1982 [35]: 12; Kirkegaard 1988 [38]: 34; Kirkegaard 1995 [37]: 41, Fig. 23; Kirkegaard 2001 [34]: 394; Arias & Paxton 2022 [39]: 3–7, Figs. 2–9.

*Hyalinoecia tubicola* var. *longibranchiata* McIntosh, 1885 [42]: 337–338 (in part).

#### Type material examined

*Hyalinoecia robusta*, BMNH 1975.194 (holotype) from off Santander, Northern Spain, RV *Sarsia*, St. 87, dredge, 43° 45.5′ N 8° 47.7.′ W, 1800 m, mud, 19.07.1968.

*Hyalinoecia tubicola* var. *longibranchiata*, BMNH 1885.12.1.231 (1 syntype) from off La Gomera, Canary Islands, HMS *Challenger*, 1097 m, 12.02.1873.

#### Material examined

North East Atlantic, off Nigeria: ZMBN 139224 (1 DNA voucher), ZMBN 91395 (1 DNA voucher), ZMBN 91393 (1 DNA voucher), ZMBN 91392 (1 DNA voucher), ZMBN 91394 (1 DNA voucher), ZMBN 156643 (5), ZMBN 156644 (1); North East Atlantic off southern Portugal: ZMBN 153529 (1 DNA voucher), ZMBN 29499 (15), ZMBN 29500 (23), ZMBN 156645 (4), ZMBN 156646 (2), ZMBN 41602 (7); North East Atlantic, east of Fuertoventura: ZMBN 29501 (2); North West Atlantic, off Massachusetts, USA: USNM 1121743 (1 DNA voucher).

#### Diagnosis

Pigmented eyespots on prostomium absent; frontal lips globular or oval, inserted frontally; branchiae from chaetiger 17–22; subacicular hooks from chaetiger 19–41; tips of anterior falcigers with 2 small rounded closely inserted teeth, covered by large hoods; tubes thick and robust almost straight.

#### Type locality

Bay of Biscay, Northern Spain, 43.783° N, 03.795° W, 1800 m.

#### Description

Holotype complete specimen 2.7 mm wide and 84 mm long divided into 2 parts: anterior part of 65 chaetigers and posterior part of 39 chaetigers; pygidium missing; tube incomplete, 116 mm long and 5 mm wide. Examined specimens 2.5–3.6 mm in width (at 10th chaetiger, without parapodia), 60–99 mm in length, all lacking posterior regions. All specimens uniform in color, light yellow or brownish lacking distinct color pattern; some specimens with darker spot on anterior margin of prostomium or with pigmented frontal lips. Prostomium rounded with paired spherical or slightly oval frontal lips, globular upper lips and wide and massive lower lip. Pigmented eyespots on prostomium absent. Ceratophores of palps and antennae with 4 (3) short rings. Palps reaching chaetiger 1 (2–3); lateral antennae reaching chaetiger 10 (30); median antenna reaching chaetiger 13 (21). Antennostyles thin and slender, tapering distally. Nuchal grooves straight, covered by anterior fold of peristomium. Peristomium slightly shorter than chaetiger 1; peristomial cirri absent.

Anterior three pairs of parapodia modified. First pair of parapodia largest, directed forward, second and third pairs smaller in size, directed anterolaterally. Subsequent unmodified parapodia directed laterally. Modified parapodia with auricular prechaetal lobes and subulate postchaetal lobes and ventral cirri. Ventral cirri become short and conical on chaetiger 4, later replaced by oval glandular pads. Postchaetal lobes remain visible, short and subulate until about chaetiger 45–65. Branchiae from chaetiger 22 (17–21) till end of body. Thick simple hooded falcigers with 2 small rounded closely inserted teeth on first pair of parapodia; often tips of falcigers broken. Second pair of parapodia with simple tapering thick and stout chaetae. Pectinate chaetae delicate with up to 20 denticles and inward rolled lateral margins from chaetiger 2. Subacicular hooks from chaetiger 32 (27–41).

Maxillary apparatus (Fig. 4a) of typical shape with short maxillary carriers, paired maxillae II, unpaired left maxillae III, paired maxillae IV and V. Maxillary formula (based on single dissected specimen from ZMBN 29500): MI = 1 + 1; MII = 14 + 14; MIII = 15 + 0; MIV = 10 + 10; MV = 1 + 1.

Tubes thick and robust, slightly curved, yellow to amber in color with circular growth ridges and inner valves at both openings, 110–165 mm long, 5.5–7.1 mm wide at the widest end (Fig. 3a, l).

#### Biology

The reproductive biology of *H. robusta* has been recently described by Arias and Paxton [39]. The species is a simultaneous hermaphrodite with a previous adolescent male phase. Spermaducal papillae—sperm storage organs that, if detached, may act as spermatophores—were reported by Arias and Paxton [39] in the specimens from the type locality and also observed in the specimens south of Portugal and off Nigeria (Figs. 3e, d, g, k and 4b, c) in the present study. The largest papillae were filled with mature spermatozoa with elongated heads, 4–5 rounded mitochondria and flagella (Fig. 4c). Arias and Paxton classified these spermatozoa as ent-aquasperm following Jamieson and Rouse [43]. Several specimens were filled with oocytes, approximately 400 µm in diameter (Fig. 3j). The species was reported to brood their young inside parental tubes [39], however no brooding specimens were observed in the present study.

#### Distribution

East Atlantic from the Bay of Biscay to Angola, depth range 440–2300 m, mostly at depths below 1000 m; West Atlantic, off Massachusetts, 1480 m; Indian Ocean, western India, off Goa, 1000 m.

## Discussion

Molecular analyses revealed five clades corresponding to four previously known species names and a single lineage herein referred to as *Hyalinoecia* sp. indistinguishable by morphology from *H. tubicola*. The detailed investigation of the newly discovered lineage is not in the focus of the present study and the formal description of a potentially cryptic species is currently under consideration.

Average uncorrected p-distances in mitochondrial markers between the five lineages varied between 6 and 18% indicating a high degree of divergence, comparable to those reported in other onuphid annelids [44, 45]. The p-distances between the lineages of *Hyalinoecia tubicola* and *Hyalinoecia* sp. were the lowest among all the species in the study (Additional file 1), which may indicate a relatively recent splitting event. The p-distances of nuclear markers were generally lower with ribosomal RNA being most conservative. Nevertheless, except for 18S gene tree, which was unresolved due to too little variation, all other gene tree topologies were congruent in recovering the five lineages discussed in the study.

Examined specimens of *Hyalinoecia longibranchiata* were collected close to the type locality, off Cape Farewell, northwestern South Island, New Zealand, at a depth of 275 m [42], and clustered together with published records of this species from the east and west of New Zealand at depths of 400–750 m [46]. A sister relationship of the *H. tubicola* complex and *H. longibranchiata* is also supported by slender tubes and the presence of pigmented eyespots on the lateral sides of prostomium.

Genetic data and the geographical records of *Hyalinoecia artifex* were obtained from Meyer et al. [30] who based their identification on the detailed re-description provided by Mangum and Rhodes [47] as the original description was lacking morphological details which became relevant for current taxonomy [41]. *Hyalinoecia robusta* is sister to *H. artifex*, although with poor support, sharing the large size of the adult specimens and the lack of dark pigmented eyespots on their prostomium.

**Fig. 5:**
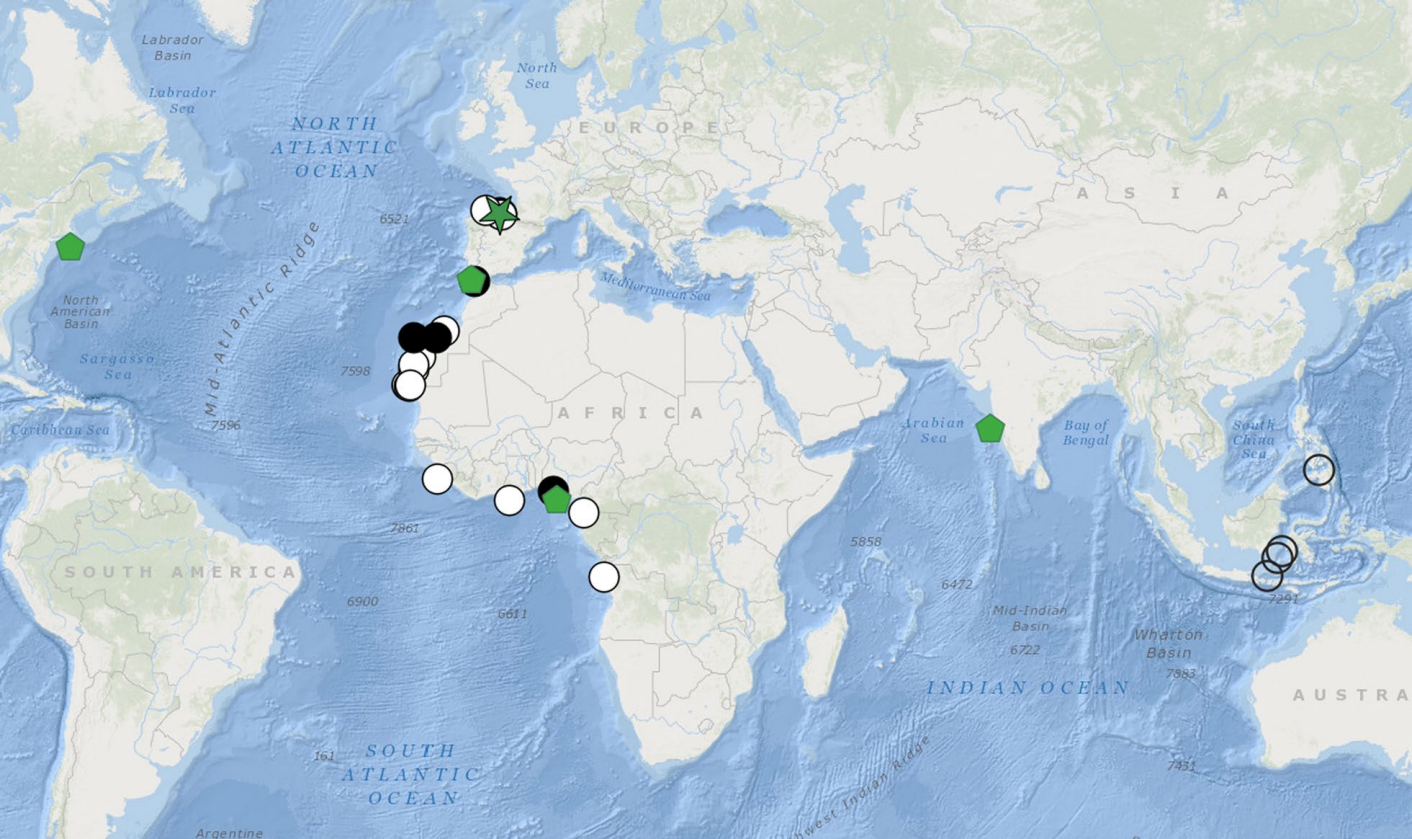
Distribution of *Hyalinoecia robusta*. Green star = type locality; green pentagons = specimens with molecular data; black circles = studied material; white circles = records from literature [33–39]; open circles = questionable records [37]. Numbers represent depths

Four *Hyalinoecia* lineages analysed in the present study seem to have localized geographical distributions limited to shelf or slope areas within single ocean basins (Fig. 2). This could represent a sampling artifact, but *Hyalinoecia* quill worms are very large and conspicuous annelids and most probably are relatively well sampled, especially in shallow waters. Although *H. tubicola* was reported from worldwide localities, no molecular data are available from the localities outside the Eastern Atlantic. Moreover, the supposed record of *H. tubicola* from the Indian Ocean was placed into the *H. robusta* clade using molecular data, suggestive of misidentification of the specimen. In contrast, *Hyalinoecia robusta* shows one of the very few examples of a widely distributed deep-sea annelid species confirmed by molecular data, with its range spanning from the western Atlantic to the Indian Oceans. The range expansion beyond the Atlantic is based on two records of *H. robusta* which were obtained from GenBank; one derived from an unidentified *Hyalinoecia* specimen used in a phylogenetic study of Eunicidae [48], and another from a specimen identified as *H. tubicola* in a barcoding project of polychaetes from West India (P. Priyaja, pers comm.). These records significantly expanded the geographical range of *H. robusta,* while the newly collected samples off Nigeria and the historical material obtained close to the type locality confirmed the range along the western African coast reported in previous studies. The records of *H. robusta* from the slope depths of Indonesia remain questionable due to a lack of molecular data and detailed morphological description of the specimens reported by Kirkegaard [37]. However, more material collected from this area may become available in the future and may allow for expanding its range even to the Pacific Ocean (Fig. 5).

The presence of long-lived pelagic larval stages in their life cycles were often considered as an explanation for long-distance dispersal capacity in marine organisms [49, 50]. Nevertheless, larval transport modelling of deep-sea invertebrates demonstrates that the actual geographical ranges may significantly exceed the presumed dispersal potential of larvae [51, 52]. One of the possible explanations for such wide ranges is the presence of unsampled populations connecting the known populations and maintaining gene flow across the range of a species. As shown in this study, *H. robusta* may be an example for such a case. Sampling slope depths along the Mid-Atlantic Ridge, the eastern African coast, the Arabian Sea or at the Atlantic and Indian Ocean seamounts could aid in discovering new populations of *H. robusta* sustaining gene flow between the populations known to date.

Confirmed pan-oceanic and trans-oceanic distributions in deep-sea annelids were reported only in a few studies [13–15, 19, 53]. *Osedax rubiplumus* Rouse, Goffredi and Vrijenhoek [54], the bone eating worm inhabiting carcases of dead whales, is known to have large-sized pelagic lecithotrophic larvae [55] aiding in the species’ dispersal capacity [53]. Among the linages sharing identical or very similar haplotypes between the Atlantic and Pacific abyssal areas, three belonged to the family Spionidae. Although the exact reproduction modes in these lineages were unknown, the species from the family Spionidae often have long-lived pelagic larvae which could aid in species dispersal over large distances but probably could not maintain the connectivity between the sampled populations > 4000 km apart [15]. Arias and Paxton [39] described the reproduction mode in *H. robusta* specimens collected close to the type locality. The worms brood their young attached to the body segments inside the parental tubes assuming no free-swimming larval stages. Direct development combined with brooding is a very common strategy in onuphid worms [56] including species of *Hyalinoecia* [57, 58]. Arias and Paxton [39] suggested that *H. robusta* has limited dispersal capacity due to its complex reproductive and brooding strategy. They proposed that the species has a limited distribution range with numerous records reported from the eastern Atlantic, and that specimens reported from the Pacific Ocean were misidentified and likely represent cryptic lineages and *H. robusta* therefore forms a species complex.

Our data do not corroborate the conclusions of Arias and Paxton [39] providing evidence of a wide distribution of *H. robusta* in the slope depths (440–2300 m) of both the Atlantic and the Indian Oceans. Our results further contradict the assumption that reproduction mode can be a good predictor of a species’ dispersal potential. Discordance between reproduction strategy and larval types on the one hand and dispersal capacity of a species on the other hand was reported in shallow-water molluscs [59] and corals [60, 61] suggesting more complex explanations are required for as species’ dispersal ability such as a combination of historical events, behavioral traits and abiotic factors.

Meißner et al. [19] suggested sediment translocations in deep sea as one of the plausible explanations for the large-scale dispersal in marine annelids. Following Hollister et al. [62], large volumes of sediment can be transported over long distances during and after abyssal storms. The sediments move together with associated benthic fauna supporting connectivity between populations in deep sea [19] even for the species lacking pelagic larvae as the main dispersal stage in their life cycle.

*Hyalinoecia robusta* are reaching up to 10 cm in length and therefore, dispersal of adult individuals with the suspended sediments over large distances appear unlikely. Nevertheless, all quill worms have epibenthic lifestyles, and their fertilized eggs and juvenile stages, supported by large amounts of yolk, could be dispersed along with moving sediments. Some onuphid juveniles are known to utilize yolk deposits until reaching up to 17–28 chaetiger stage [56, 63]. In *Diopatra aciculata* Knox & Cameron, 1971 [64], the worms reach the size of 14–15 chaetigers in 28–35 days. Although feeding in this species starts at a much earlier stage of 5 chaetigers, it can indicate the general growth rates in onuphid worms [65]. Although *Hyalinoecia* do not have a long-lived feeding pelagic stage, their non-feeding lecithotrophic juveniles have a potential for long-distance dispersal through sediment translocation.

Our results further highlight the importance of curated open databases with molecular information linked to vouchered specimens deposited in natural history collections. Recent studies successfully obtaining DNA barcode sequence information from archival invertebrate wet-collection specimens using a combination of ancient DNA methodology and target capture [25, 66–68] are generally effective to add molecular information for aged museum specimens not sequenced so far [69, 70]. We successfully included a wet-collection specimen of 113 years of age in our sampling overcoming the challenges associated with DNA sequencing of such samples. In this initial experiment, a large amount of tissue was available, however, the amount of DNA needed for successful single stranded library constructions can be lower as detailed in [26]. Depending on the fixation and preservation history, less amounts of tissue may still result in successful DNA sequencing. Our study exemplifies that the usage of genetic information from museum material should become standard to extend the available genetic basis for biogeographic studies of deep-sea annelids. This approach allows for more accurate evaluation of distribution ranges of species providing the basis for conservation efforts.

Moreover, ancient DNA methods open an opportunity for obtaining molecular information from type materials, which is especially needed for those species described from the nineteenth century based on syntype series, i.e. numbers of specimens often collected from several distant localities. Early species descriptions are often very brief and selection of a lectotype with associated molecular data can stabilize the use of names and clarify the geographical ranges of species.

## Conclusions

Our study highlights the significance of curated open access databases with molecular information preliminarily linked to vouchered specimens deposited in scientific collections. In combination with DNA sequence information of aged museum samples, obtained applying ancient DNA methodology, our study allows for re-analysing the distribution patterns of five *Hyalinoecia* lineages. While our results confirm limited local geographical distributions for four lineages, *H. robusta* shows a wide distribution range spanning from the western Atlantic to the Indian Ocean. We demonstrate that the reproductive mode of *H. robusta* is not limiting large-scale dispersal, challenging the assumption that a species’ reproductive strategy is indicative for its dispersal ability.

## Materials and methods

### Taxon sampling

We studied 99 specimens of *Hyalinoecia* worms in total representing two species known from the Northeast Atlantic: *H. robusta* and *H. tubicola* as well as *H. longibranchiata* from the Southern hemisphere; 39 specimens were sampled for molecular analyses.

The recent material included the samples from *H. tubicola* and *H. robusta* collected during the series of RV *Dr. Fridtjof Nansen *expeditions in 2005–2012 as a part of the EAF-Nansen programme; the DIVA-3 expedition (Latitudinal Gradients in BioDIVersity in the deep Atlantic, 2009); an environmental monitoring survey (2011); and in a coastal sampling trip (2004) by the University of Bergen in the North Sea (Additional file 2). The specimens were preserved in 96% ethanol at + 4 °C.

Historical material included specimens of *H. robusta* and *H. longibranchiata.* Fifty-four specimens of quill worms obtained during the RV *Michael Sars* North-Atlantic Deep-Sea Expedition 1910 [40] were examined at the invertebrate collection of the University Museum of Bergen. The deep-water (1365–2055 m) specimens originally identified as *Hyalinoecia tubicola* appeared to be much larger in size compared with specimens sampled from the shelf areas. One of the specimens from ZMBN 29500 collected on 8 May 1910 at a depth of 2055 m south of Portugal was selected for DNA barcoding using hybridization capture. In the present study, this specimen was assigned the new catalogue number—ZMBN 153529. Five specimens of *H. longibranchiata* collected on 12 January 1976 by the Soviet Expedition on board of RV *Dmitry Mendeleev* to the Southern Pacific (Additional file 2) originally identified as *Hyalinoecia tubicola* were borrowed from the collection of the Shirshov Institute of Oceanology, Russian Academy of Sciences. The original preservation liquid is unknown, however, we successfully amplified most of the targeted genetic markers from five specimens that were stored at room temperature for about 40 years using polymerase chain reactions (Additional file 2).

Several sequences were additionally obtained from the GenBank sequence database. These included the sequences of 16S of *Hyalinoecia artifex*, a species known from the Northwest Atlantic [30]; COI, 16S and 18S sequences derived from a single *Hyalinoecia* sp. specimen collected off Massachusetts [48]; and a COI sequence of *H. tubicola* obtained from off western India (P. Priyaja, pers. comm.). We additionally included GenBank deposited sequences of COI and 16S of *Hyalinoecia longibranchiata* from off New Zealand [46] and previously published sequences of COI, 16S, 28S and 18S of *H. tubicola* and *Hyalinoecia* sp. [25, 71, 72] (Additional file 2).

Four representatives of the genera *Nothria*, *Leptoecia*, and *Australonuphis* were used as outgroups in the phylogenetic analysis. Their sequences were either obtained from GenBank or generated during this study from the same voucher specimens (Additional file 2). The sequences used in this study are derived from the specimens deposited in the following museum collections: University Museum of Bergen, University of Bergen, Norway (ZMBN), Senckenberg Museum Frankfurt, Germany (SMF), National Museum of Natural History, Smithsonian Institution, USA (USNM), Australian Museum, Sydney, Australia (AM), NIWA Invertebrate Collection, New Zealand (NIC). The type materials of *Hyalinoecia robusta* are stored in the Natural History Museum London, UK (BMNH).

### Morphology

The specimens were examined and identified using light and scanning electron microscopy (SEM). For SEM, the specimens were dehydrated using a gradient series of ethanol—hexamethyldisilazane (HMDS) mixtures with at least 2 h at each step: 100% EtOH, 75% EtOH–25% HMDS, 50% EtOH–50% HMDS, 25% EtOH–75% HMDS, 100% HMDS. After that the specimens were left under a fume hood in 100% HMDS until completely dried, mounted on SEM stubs, coated with gold, and examined with a ZEISS Supra 55VP scanning electron microscope at the Laboratory for Electron Microscopy, University of Bergen. Measurements of width were taken at the level of the 10th chaetiger excluding parapodia. Examination of jaws was done by dissecting them from the muscular pharynx via dorsal longitudinal incision. Male gametes were examined by cracking the dorsal papillae with forceps in dehydrated specimens. Terminology follows Budaeva et al. [71]. 

### Sanger sequencing

Genomic DNA was extracted using the QIAGEN DNeasy® Blood & Tissue Kit. Four nuclear (18S, 28S, ITS1 and ITS2) and two mitochondrial (COI and 16S) markers were amplified using the primers and PCR protocols listed in Additional file 3. Amplification of the targeted regions was performed using the TaKaRa^®^ Ex Taq HS kit. The final reaction of 25 µl consisted of 1 µl of DNA template, 17.35 µl of purified water, 2.5 µl of 10 × Ex Taq buffer, 2 µl of dNTP mixture, 1 µl of each primer and 0.15 µl of TaKaRa^®^ Ex Taq HS. PCR products were purified using ExoSAP-IT. Sequencing reactions for both strands of the amplified fragments were performed using the BigDye Terminator v3.1 Cycle Sequencing Kit (Applied Biosystems) with the same primers as for PCR. Products were sequenced using an Applied Biosystems automated sequencer. Sequence contigs were assembled and edited in Sequencher v. 4.5 (Gene Codes, Ann Arbor, Michigan).

### Historical material high throughput sequencing

DNA extraction steps, single stranded library preparation, and test-sequencing including the evaluation of the library’s sequence content of sample ZMBN 153529 are detailed in Agne et al. [25]. In summary, 50 mg tissue was used for DNA extraction applying the guanidine approach [26] which is based on Dabney et al. [73] and Rohland et al. [74]. The tissue sample comprised several posterior segments allowing for testing the extraction method on the upper limit of tissue still useful in the extraction. Thirteen nanogram of DNA were subsequently used in the single stranded library construction following [75] which represents the upper limit of DNA amount to be inserted. Test-sequencing was performed as described in [26] to check for the ratio of target DNA and contamination. Target capture was performed using the customized mixed RNA bait set as described in Agne et al. [25]. This myBaits^®^ kit (Arbor Biosciences, Ann Arbor, Michigan, USA) contained RNA baits covering multiple different markers used for species barcoding approaches of diverse animals including baits designed from sequence information of two mitochondrial (COI, 16S) and four nuclear (ITS1, ITS2, 28S, 18S) markers derived from *H. tubicola* as described in Agne et al. [25] to obtain DNA sequence information from the archival wet-collection specimen ZMBN 153529. Target capture was performed twice following the protocol by Huang et al. [76] including two rounds of amplification after evaluation of the optimal number of amplification cycles to avoid over-amplification of adapters. Test sequencing and subsequent sequencing of the captured libraries was performed on an Illumina NextSeq 500 System at the University of Potsdam as described in Paijmans et al. [77]. Raw sequencing reads were quality filtered and adapters trimmed with Cutadapt v. 2.10 [78]. Next, sequencing reads were mapped against the bait sequences, i.e., the six loci used as bait sequences. For this, BWA-ALN v. 0.7.17 [79] was used. After mapping, PCR duplicates were removed with Samtools v. 1.10 and consensus sequences called using Bcftools v. 1.9 [80]. The consensus sequences were then added to the alignment step.

### Alignment and phylogenetic analysis

Sequences were concatenated using PhyloSuite v. 1.2.2. [81] and aligned with MAFFT v. 7.453 [82, 83]. For the final alignment (4926 bp), best-fitting substitution models were estimated using Modelfinder and the Akaike information criterion (AIC) as implemented in PhyloSuite. A Bayesian inference-based phylogeny was computed using MrBayes v. 3.2.7a [84] under the GTR + F + I + G4 substitution model, which considers base frequencies directly from the alignment and allows for a proportion of invariable sites and a discrete Gamma model. The analysis was run for 5,000,000 generations. Trees were sampled every 1000th generation and 25% of 10,002 sampled trees discarded as burn-in. Two runs and 4 chains were run in parallel and checked for convergence using Tracer v. 1.7.2 [85]. The alignment was further used for a second, maximum likelihood-based approach using IQ-TREE v. 2.0.3 [86] applying the general time reversible model substitution. Taxa *Nothria* sp., *Nothria conchylega*, *Leptoecia midatlantica*, and *Australonuphis teres* were defined as outgroups in both analyses.

### Distances

Average evolutionary distances (p-distances) were computed within each species clade and between species using MEGA11 [87]. All positions with less than 95% site coverage were eliminated. That is, fewer than 5% alignment gaps, missing data, and ambiguous bases were allowed at any position (Additional file 1).

### Supplementary Information

**Additional file 1**. Uncorrected pairwise within- and between clade distances of the markers analyzed in this study (COI, 16S rRNA, 18S rRNA, ITS1, ITS2, and 28S rRNA).

**Additional file 2**. List of specimens used in this study with voucher numbers, accession numbers, BOLD process IDs and locality data. Accession numbers for the sequences obtained in this study are marked in bold.

**Additional file 3**. Primers and PCR settings for the markers analyzed in this study (COI, 16S rRNA, 18S rRNA, ITS1, ITS2, and 28S rRNA).

## Data Availability

All physical specimens used in this study are kept in the registered museum collections. All sequences have been submitted to GenBank and BOLD (see Additional file 2 for accession numbers and BOLD process IDs).
